# Ursodeoxycholic Acid Alters Bile Acid and Fatty Acid Profiles in a Mouse Model of Diet-Induced Obesity

**DOI:** 10.3389/fphar.2019.00842

**Published:** 2019-07-25

**Authors:** Yunjing Zhang, Xiaojiao Zheng, Fengjie Huang, Aihua Zhao, Kun Ge, Qing Zhao, Wei Jia

**Affiliations:** ^1^School of Biomedical Engineering, Shanghai Jiao Tong University, Shanghai, China; ^2^Shanghai Key Laboratory of Diabetes Mellitus and Center for Translational Medicine, Shanghai Jiao Tong University Affiliated Sixth People’s Hospital, Shanghai, China; ^3^Cancer Biology Program, The University of Hawaii Cancer Center, Honolulu, HI, United States

**Keywords:** ursodeoxycholic acid, bile acid, free fatty acid, diet-induced obesity, metabolic dysfunction, traditional Chinese medicine

## Abstract

Ursodeoxycholic acid (UDCA) is a bile acid (BA) approved by the U.S. Food and Drug Administration for the treatment of primary biliary cholangitis. It is also the major active component of bear bile used in traditional Chinese medicine to reduce fever, remove toxins, and treat liver and eye ailments. In addition, UDCA and its conjugated form have been evaluated for their potential to improve symptoms of metabolic diseases, but the results have been inconclusive. To address this issue, in this study, we investigated the effects of orally administered UDCA on mice with diet-induced obesity, including the BA and free fatty acid (FFA) profiles of serum, liver, and epididymis and brown adipose tissues. We found that UDCA treatment significantly improved most metabolic indices; tauroursodeoxycholic acid (TUDCA) and taurolithocholic acid (TLCA) contents were increased in all examined tissues, whereas saturated FA levels were decreased, and n-3 polyunsaturated fatty acid (n-3 PUFA) levels were increased in most tissues. A correlation analysis showed that the concentrations of UDCA and its derivatives were positively correlated with that of n-3 PUFA. To clarify the mechanism by which UDCA alters FFA profiles, we analyzed the expression levels of genes involved in FFA biosynthesis, uptake, and oxidation and found that FFA biosynthesis and uptake were inhibited while FFA oxidation was stimulated by UDCA treatment. Additionally, amino acid-conjugated derivatives of UDCA, such as TUDCA and TLCA, altered FFA profiles by modulating FFA biosynthesis, uptake, and oxidation. These findings provide evidence that UDCA can alleviate metabolic dysfunction and could therefore be effective in the treatment of obesity.

## Introduction

Obesity is a major risk factor for the development of type 2 diabetes, nonalcoholic fatty liver disease (NAFLD), and cardiovascular disease (CVD). Approximately, 35% of adult men and 37% of women in the U.S. are obese (Yang and Colditz, 2015). Elevated plasma free fatty acid (FFA) level is observed in obesity, which is thought to play a key role in the progression of obesity-associated insulin resistance and CVD (Boden, 2011). We previously demonstrated that plasma FFA levels are reliable markers for predicting metabolic abnormalities in obese individuals (Ni et al., 2015; Zhao et al., 2016; Zhao et al., 2017), and normalizing plasma FFA levels has been proposed as a potential therapeutic strategy for the treatment of obesity and metabolic diseases (Boden and Shulman, 2002). Accumulating evidence suggests that bile acid (BA) plays a role in metabolic diseases and affects sensitivity to insulin, which regulates glucose levels, lipid homeostasis, and energy expenditure* via *activation of BA receptors in the liver, gut, and peripheral tissues (Kuipers et al., 2014; Schaap et al., 2014; Kanwal et al., 2018). Circulating BA levels are associated with NAFLD and are correlated with histological features of nonalcoholic steatohepatitis (NASH) (Puri et al., 2018). Additionally, circulating BA and FFA levels are thought to influence each other (Kim et al., 2017; Liu et al., 2017; Lu et al., 2017), although the mechanism underlying this interaction is unclear.

Ursodeoxycholic acid (UDCA) is a BA with choleretic, anti-inflammatory, and cytoprotective properties (Paumgartner and Beuers, 2004) that has been approved by the U.S. Food and Drug Administration for the treatment of primary biliary cholangitis (Marschall et al., 2005). UDCA is also the major component of bear bile, which is used in traditional Chinese medicine (TCM) to reduce fever, remove toxins, and treat liver and eye ailments. In recent years, accusations of animal cruelty have been leveled against TCM manufacturers and drug companies that produce medicines from bear bile. By clarifying the biochemical and pharmacological effects of UDCA, it may be possible to develop UDCA-based treatments that can substitute or minimize the use of bear bile in TCM.

The therapeutic potential of UDCA and its conjugated form in metabolic diseases has been evaluated in multiple studies. However, UDCA has shown contradictory effects in NAFLD. One study found a reduction in steatosis (Laurin et al., 1996), while randomized placebo-controlled trials reported no improvement in ballooning and inflammation in patients with NASH (Lindor et al., 2004; Leuschner et al., 2010). A short-term study of UDCA showed that it increased hepatic triglyceride (TG) content (Mueller et al., 2015). On the other hand, taurine-conjugated (T)UDCA reduced hepatic steatosis and enhanced the activity of insulin in mouse liver, muscle, and adipose tissues (Ozcan et al., 2006) as well as hepatic and muscle insulin sensitivity in obese subjects (Kars et al., 2010), while glycine-conjugated (G)UDCA improved metabolic parameters in a mouse model of obesity (Sun et al., 2018). Thus, the effects of UDCA on metabolism and its role in metabolic disease warrant further investigation.

To address this issue, the present study evaluated the effects of orally administered UDCA in mice with diet-induced obesity by analyzing BA and FFA profiles in serum, liver, epididymis adipose tissue (EAT), and brown adipose tissue (BAT). We also examined the relationship between BAs and FFAs as well as the expression of genes involved in lipogenesis, lipid oxidation, and FA transport in UDCA-treated obese mice.

## Materials and Methods

### Chemicals and Reagents

The control diet fed to mice contained 10% lipid, 19% protein, and 71% carbohydrate. The high-fat diet contained 60% lipid, 19% protein, and 21% carbohydrate (Trophic Animal Feed High-tech Co., Nantong, China). UDCA used in this experiment was purchased from J&K Scientific (catalog no. 970735; lot no. LK60R25; Shanghai, China) and its purity was about 94% which had been detected by UPLC-QTOF-MS, and the chromatogram of the dominating compounds was shown in **Supplementary Figure 1** and the structure of UDCA was shown in **Supplementary Figure 2**. The 54 FFA standards were obtained from Sigma-Aldrich, and the 49 BA standards were from Sigma-Aldrich or Steraloids (Newport, RI, USA). Primers for quantitative real-time (qRT-)PCR were synthesized by Sangon Biotech (Shanghai, China) and used along with TRIzol reagent (Invitrogen, Carlsbad, CA, USA), Prime Script RT Reagent Kit (Takara Bio, Otsu, Japan), and Power Up SYBR Green PCR Master Mix (Thermo Fisher Scientific, Waltham, MA, USA).

### Animal Experiments

This study was carried out in accordance with the recommendations of the national legislation and local guidelines of the Laboratory Animals Center at Shanghai Jiao Tong University Affiliated Sixth People’s Hospital, Shanghai, China. The protocol were reviewed and approved by the Institutional Animal Care and Use Committee at the Center for Laboratory Animals, Shanghai Jiao Tong University Affiliated Sixth People’s Hospital, Shanghai, China.

Specific pathogen-free male C57BL/6 mice (17.44 ± 0.80 g; 4 weeks old) were purchased from SLAC Laboratory (Shanghai, China) and housed under temperature-controlled (22–25°C) specific pathogen-free conditions on a 12:12-h light/dark cycle (with lights on at 7:00 a.m.) with free access to food and water. Mice were allowed to acclimate to the housing facility for 1 week before they were used for experiments.

The mice were randomly divided into three groups (*n* = 6 each) that were fed a normal chow diet (control), high-fat diet (HFD; 60% fat), and HFD with 0.5% UDCA (w/w). After 8 weeks, the mice were fasted for 12 h and sacrificed, and serum, EAT, and BAT were collected and the liver was removed. The samples were frozen in liquid nitrogen and stored at −80°C until use.

### Oral Glucose Tolerance Test (OGTT) and Insulin Tolerance Test (ITT)

The OGTT and ITT were performed on mice after 7 weeks on the specific diet. For the OGTT, 1 g/kg of glucose was administered by oral gavage and for the ITT and 0.75 U/kg of insulin was administered by intraperitoneal injection. Blood samples were collected from tail vein 0, 15, 30, 60, and 120 min later, and glucose levels were measured with a glucometer (Johnson & Johnson, New Brunswick, NJ, USA).

### Evaluation of Biochemical Parameters and Hepatic Lipid Levels

Serum alanine aminotransferase, aspartate aminotransferase, total cholesterol (TC), TG, and high- and low-density lipoprotein (LDL) levels were measured with a Chemray Automatic Biochemical Analyzer (Shenzhen Redu Life Technology, Shenzhen, China). Hepatic lipids were extracted by the Folch method. Briefly, liver tissue was homogenized with a chloroform/methanol (2:1 [v/v]) solution to a final volume 20 times that of the tissue sample followed by dispersion, agitation, and centrifugation. The various biochemical parameters were measured according to the manufacturer’s protocol.

### BA Analysis

BA in serum and liver tissue was extracted and quantified as previously described (Xie et al., 2013). Briefly, samples were weighed and sequentially extracted with a mixture of methanol:water (1:1 [v/v]) and methanol:acetonitrile (2:8 [v/v]) by homogenization and centrifugation. Six internal standards [d4-lithocholic acid (LCA), d4-UDCA, d4-cholic acid (CA), d4-glycodeoxycholic acid (GDCA), d4-glycocholic acid (GCA), d4-deoxycholic acid (DCA)] were added to the extraction solution (50 nM). The supernatants from the two extraction steps were combined, and BA level was quantified by ultra-performance liquid chromatography–triple quadruple mass spectrometry (UPLC-TQ-MS) (Waters, Milford, MA, USA). The BA in EAT and BAT was extracted as described above for the first two steps, and then hexane was added to the combined supernatant to remove lipids. The liquid under the hexane layer was transferred to a new tube and dried under vacuum, and reconstituted with a mixture of methanol, water, and acetonitrile for UPLC-TQ-MS analysis.

The column mobile phase consisted of water (solvent A) and acetonitrile/methanol (87:13 [v/v], solvent B). An Acquity ethylene bridged hybrid (BEH) C18 column (2.1 ×100 mm, 1.7-μm internal diameter; Waters) was used at a flow rate of 0.45 ml/min and column temperature of 45°C. The gradient elution conditions were shown in the **Supplementary Table 1**. The mass spectrometer was operated with an electrospray ionization (ESI) source, and the analysis was carried out in the negative mode with a capillary voltage of 2.5 kV, source temperature of 150°C, and desolvation temperature of 500°C. The cone and collision voltages of individual BAs were described in detail in the **Supplementary Table 2**. A standard calibration solution with 49 BA standards at 14 different concentrations was used to generate the calibration curve by internal standard adjustment with the TargetLynx application (Waters).

### FFA Analysis

FFAs in serum and other tissues were extracted and quantified as previously described (Zheng et al., 2016). Briefly, samples were weighed and extracted with a mixture of isopropanol and hexane containing phosphate by homogenization and centrifugation. The supernatant was mixed with internal standard (nonadecylic acid-D37) and then extracted with hexane and water. The mixture was transferred to a new tube and dried under vacuum and reconstituted with methanol for UPLC-QTOF-MS analysis.

The column mobile phase consisted of water (solvent A) and acetonitrile/isopropyl (80:20 [v/v], solvent B). An Acquity BEH C18 chromatographic column was used for separation at a flow rate of 0.4 ml/min and column temperature of 40°C. The gradient elution conditions were shown in the **Supplementary Table 3**. The mass spectrometer was operated with an ESI source, and the analysis was carried out in the negative mode with a capillary voltage of 2.5 kV, sampling cone at 55 V, extraction cone at 4 V, source temperature of 150°C, and desolvation temperature of 450°C. A standard calibration solution with 54 FFA standards at 11 different concentrations was used to generate the calibration curve. Peak annotation and quantitation were carried out using TargetLynx.

### Quantivite Real-Time PCR

Total RNA was isolated from frozen tissue using TRIzol reagent according to the manufacturer’s instructions. RNA concentration was measured with a NanoDrop 2000C spectrophotometer (Thermo Fisher Scientific). Reverse transcription was performed using the RT Reagent Kit (Takara Bio) according to the manufacturer’s instructions, and qRT-PCR was performed on an ABI 7900 instrument (Applied Biosystems, Foster City, CA, USA). The expression levels of target genes were determined relative to that of *glyceraldehyde 3-phosphate dehydrogenase* mRNA.

### Statistical Analysis

Results are expressed as mean ± SEM of at least three independent experiments. Mean differences between two groups were evaluated with the unpaired two-tailed Student’s *t* test. Correlations between BA and FFA levels were assessed with Spearman’s correlation. *P* < 0.05 was considered statistically significant.

## Results

### Oral Administration of UDCA Improves Metabolic Dysfunction in Mice on HFD

**Figure 1:**
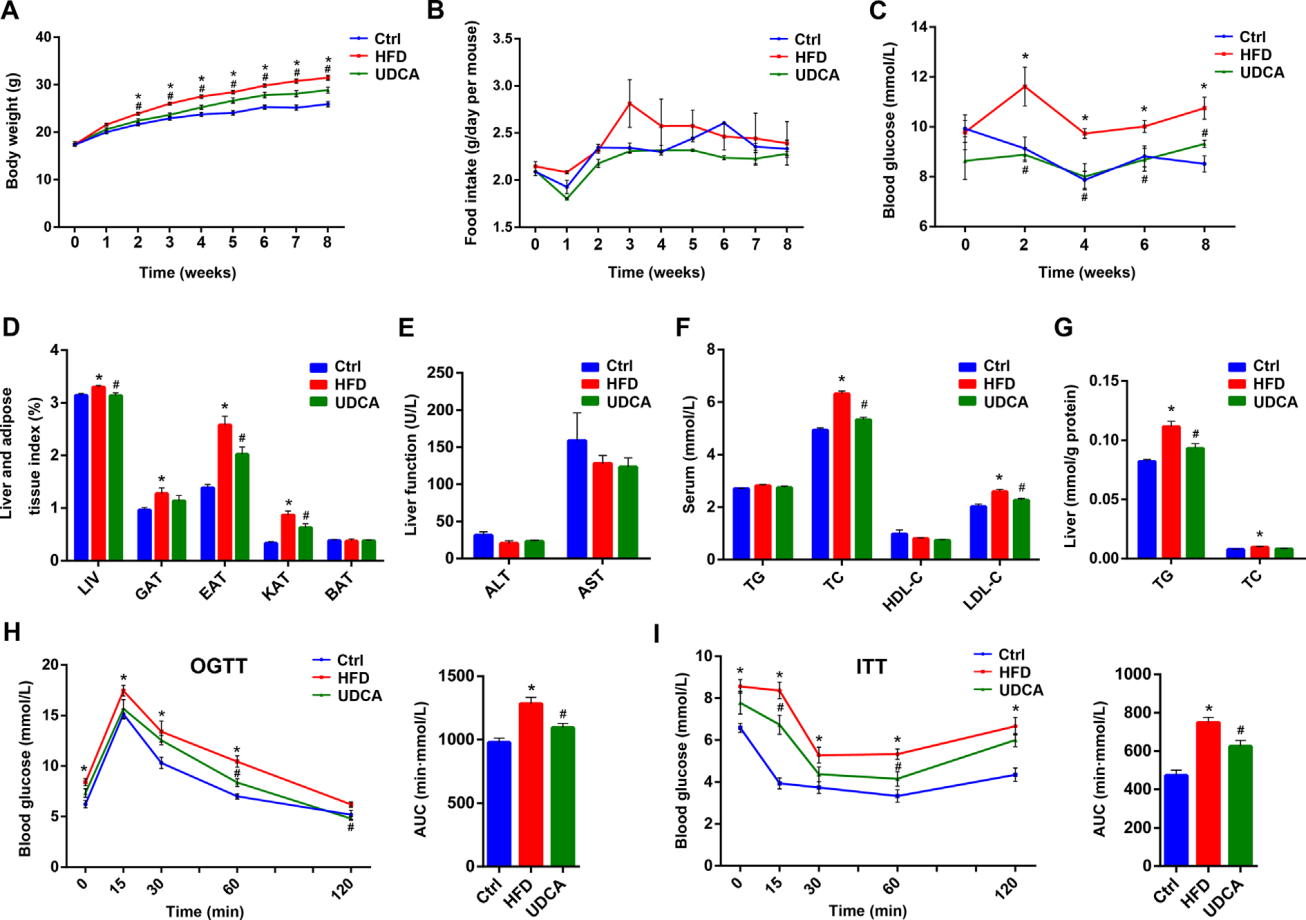
Metabolic markers 8 weeks after high-fat diet (HFD) and ursodeoxycholic acid (UDCA) administration. **(A)** Changes in body weight over time. **(B)** Food intake over time. **(C)** Blood glucose over time. **(D)** Liver and adipose tissue index at week 8. **(E)** Liver function at week 8. **(F)** Serum TG, TC, high-density lipoprotein cholesterol (HDL-C), and LDL-C levels. **(G)** Liver levels of triglyceride (TG) and total cholesterol (TC). **(H)** Oral glucose tolerance test (OGTT) curve and area under the curve at week 7. **(I)** insulin tolerance test (ITT) curve and area under the curve at week 7. Ctrl, control group; HFD, 60% fat diet; UDCA, HFD with 0.5% UDCA. *P < 0.05 *vs*. control (ctrl) group; ^#^ *P* < 0.05 *vs*. HFD group.

We compared the expression of metabolic markers in HFD mice with or without UDCA treatment to assess the effect of UDCA on obesity. Body weight was increased in the HFD group relative to control mice starting from the second week; however, mice in the UDCA group maintained a normal body weight (**Figure 1A**) that was not due to lower food intake (**Figure 1B**). A similar trend was observed for blood glucose levels (**Figure 1C**). After 8 weeks, liver and adipose tissue indices except for BAT were increased in the HFD group whereas liver index and two indices of visceral fat were decreased in the UDCA group (**Figure 1D**). There was no liver damage caused by HFD or UDCA administration after 8 weeks (**Figure 1E**). Serum TC and LDL cholesterol levels were markedly reduced in the UDCA as compared to the HFD group (**Figure 1F**). In liver, total TG was decreased after 8 weeks of UDCA treatment (**Figure 1G**) and glucose and insulin tolerance was improved (**Figure 1H, I**). Thus, UDCA alleviates metabolic dysfunction in mice with HFD-induced obesity, as evidenced by the decreases in body weight, visceral fat accumulation, and glucose level.

### UDCA Reverses Changes in BA Profile Induced by HFD

**Figure 2:**
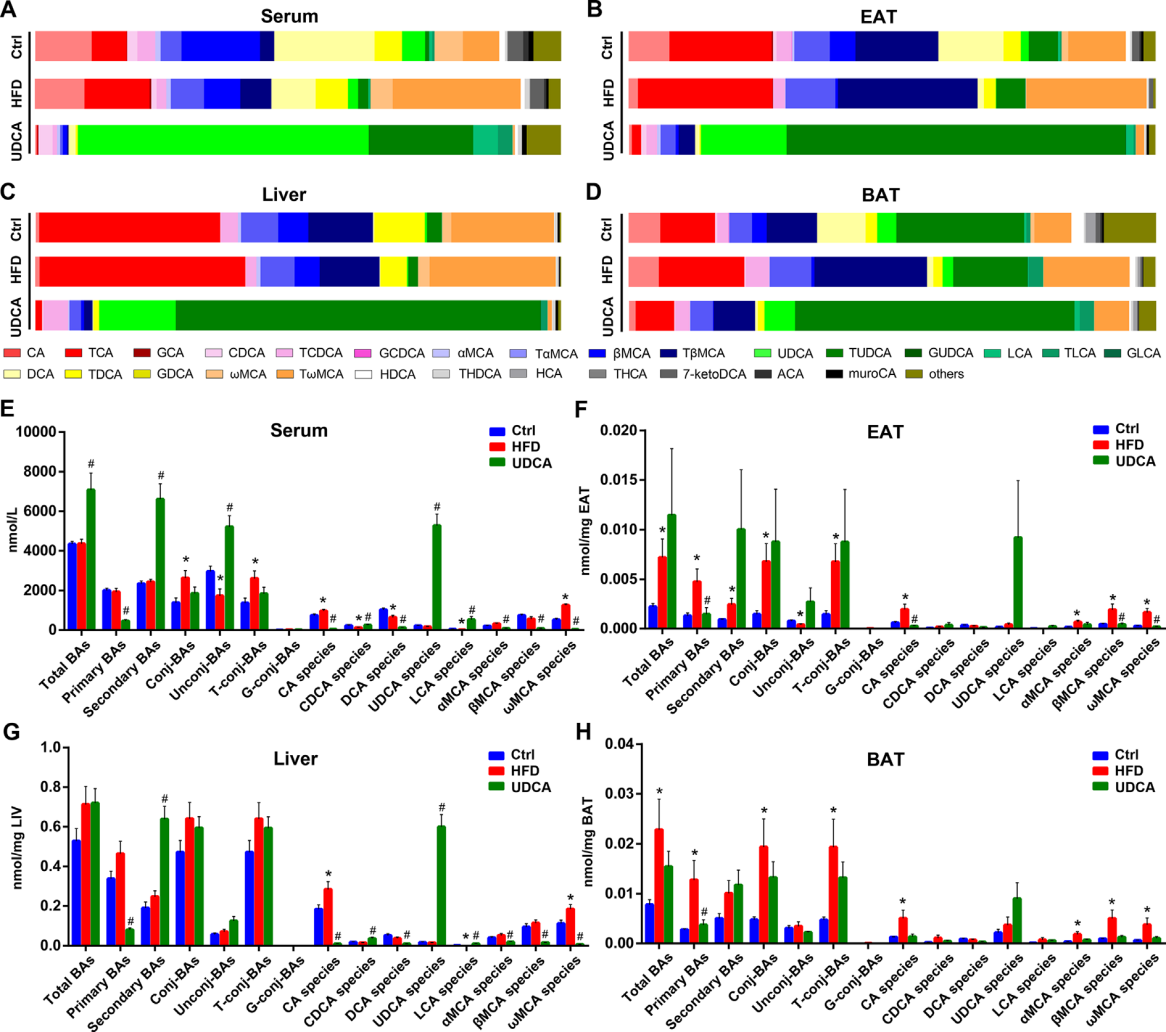
Bile acid (BA) composition of different tissues. **(A**–**D)** BA composition of serum **(A)**, epididymis adipose tissue (EAT) **(B)**, liver **(C)**, and brown adipose tissue (BAT) **(D)**. **(E**–**H)** Concentration of different BA species in serum **(E)**, EAT **(F)**, liver **(G)**, and BAT (**H**). Ctrl, control group; HFD, 60% fat diet; UDCA, HFD with 0.5% UDCA. *P < 0.05 *vs*. control (ctrl) group; ^#^ *P* < 0.05 *vs*. HFD group.

Compared to mice on a normal control diet, those in the HFD group showed altered BA composition in different tissues (**Figures 2A–D**), although total BA levels in serum and liver were not significantly changed (**Figures 2E, G**). However, total BA was increased in EAT and BAT (**Figures 2F, H**). Conjugated BA content was increased in serum and both types of adipose tissue in the HFD group (**Figures 2E–H**), whereas CA and ω-muricholic acid (ωMCA) species were increased in all tissues (**Figures 2E–H**). Additionally, serum chenodeoxycholic acid (CDCA) and DCA levels were decreased (**Figure 2E**) whereas αMCA and βMCA were increased in EAT and BAT (**Figure 2F, H**) by HFD. Meanwhile, total ωMCA was increased in all tissues in the HFD group (**Supplementary Figure 3A–D**) and TCA and GCA increased in serum, liver, and EAT (**Supplementary Figure 3A–C**). We also found that UDCA and TUDCA compositions were higher in BAT than in EAT in mice fed a normal diet and HFD (**Figures 2B, D**). Similarly, LCA, TLCA, and other UDCA derivatives such as 7-ketolithocholic acid (7-ketoLCA) were elevated in BAT relative to EAT (**Supplementary Figure 3C, D**).

Upon UDCA administration, the levels of UDCA species increased in all tissues (**Figures 2A–D**); the level of UDCA was much higher than that of TUDCA in serum, but in other tissues, TUDCA dominated the BA profile (**Figures 2A–D**). Total BA in serum was increased in the UDCA group (**Figures 2E**) whereas no changes were observed in other tissues (**Figures 2F–H**). In addition to UDCA, CDCA and LCA contents were higher in serum and liver after UDCA treatment (**Figures 2E, G**). Meanwhile, CA, DCA, α-muricholic acid (αMCA), β-muricholic acid (βMCA), and ωMCA species were decreased in the UDCA group compared with HFD group (**Figures 2E–H**), indicating that UDCA was not converted to MCAs *in vivo* with HFD and may have inhibited the production of CA and its derivatives. The levels of some other BAs in the serum and liver increased after UDCA administration, including the potential UDCA derivatives 3β-ursodeoxycholic acid (β-UDCA), 6-ketolithocholic acid (6-ketoLCA), 7-ketoLCA, dehydrolithocholic acid (dehydroLCA), and isolithocholic acid (isoLCA) (**Supplementary Figure 3A, 3B**). Thus, UDCA administration reversed the alterations in the BA profile induced by HFD by increasing UDCA and its derivatives, decreasing CA and ωMCA species contents, thereby improving metabolic symptoms.

### UDCA Reverses Changes in FA Profile Induced by HFD

**Figure 3:**
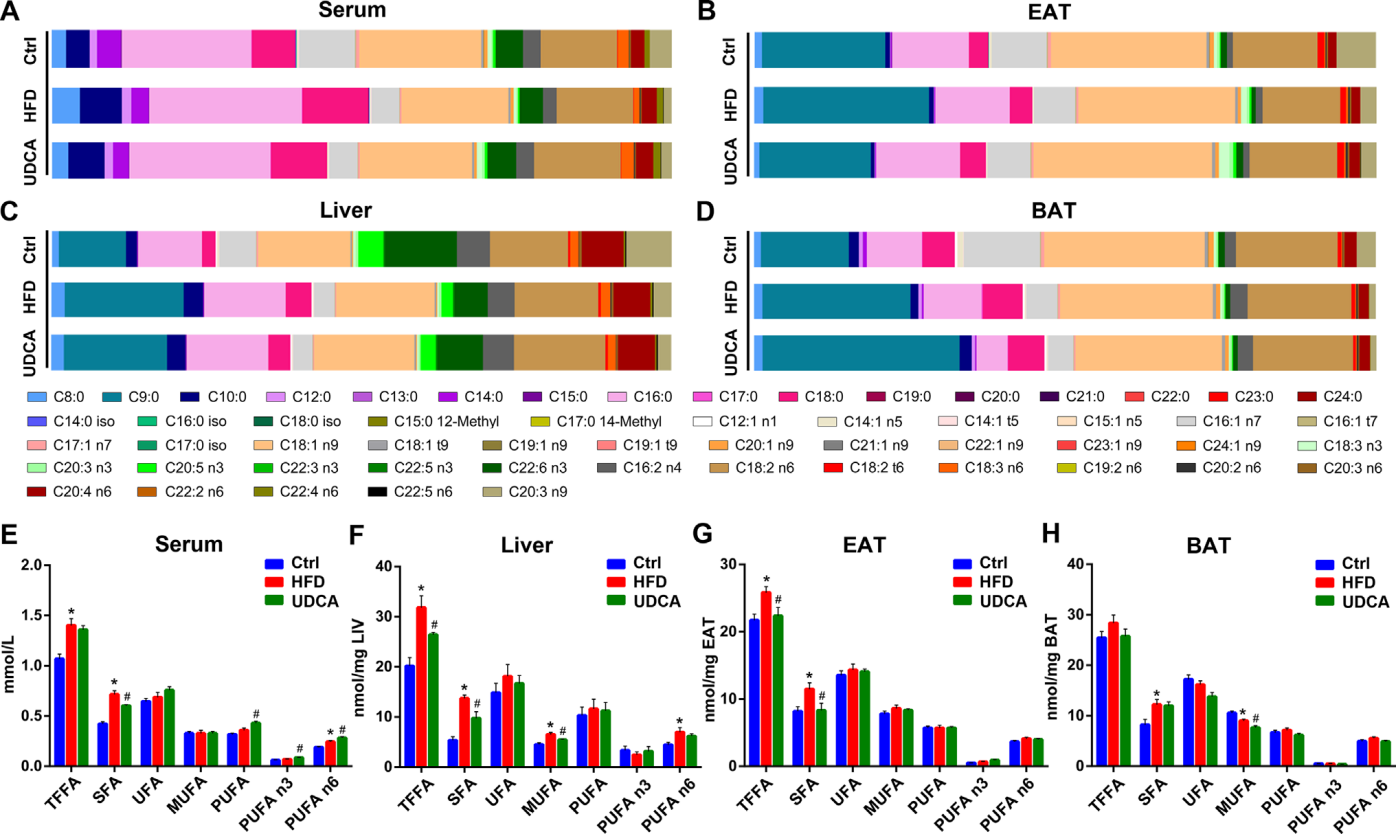
Free fatty acid (FFA) composition and concentration of FFAs in different tissues. **(A**–**D)** FFA composition of serum **(A)**, EAT **(B)**, liver **(C)**, and BAT **(D)**. **(E**–**H)** Concentration of different FFA species in serum **(E)**, liver **(F)**, EAT **(G)**, and BAT **(H)**. Ctrl, control group; HFD, 60% fat diet; UDCA, HFD with 0.5% UDCA. *P < 0.05 *vs*. control (ctrl) group; ^#^ *P* < 0.05 *vs*. HFD group.

After 8 weeks on HFD, FFA composition changed dramatically (**Figures 3A–D**). Total FFA was elevated in serum, liver, and EAT (**Figures 3E–G**), with a particularly notable increase in saturated FA (SFA) (**Figures 3E–G**). SFA was also increased in BAT, although total FFA showed no change (**Figure 3H**). Specifically, C18:0 level was higher in all tissues (**Supplementary Figure 4A–D**) and C16:0 level was higher in serum and liver of the HFD group relative to the control (**Supplementary Figure 4A, B**).

The improvement of metabolic markers and changes in the BA profile induced by UDCA was accompanied by alterations in the FFA profile. The FFA composition of the UDCA group was close to that of normal control animals after UDCA administration, except in BAT (**Figures 3A–D**). UDCA treatment reduced total FFA in liver and EAT (**Figures 3F, G**), which was consistent with the decline of liver and EAT indices (**Figure 1D**). Although there was no change in total serum FFA, there was a decrease in the levels of SFA (**Figure 3E**) especially long-chain FA (LCFA) (**Supplementary Figure 4A**). Additionally, the levels of LCFAs such as C16:0 and C18:0 were also decreased in BAT (**Supplementary Figure 4D**). Serum PUFAs such as C18:3 n3, C20:3 n3, C18:3 n6, and C20:3 n9 increased in UDCA group (**Figure 3E**, **Supplementary Figure 4A**). After UDCA treatment, the levels of some monounsaturated FAs (MUFAs) such as C18:1 n9, C20:1 n9, and C22:1 n9 declined in liver and BAT (**Supplementary Figure 4B, D**). In summary, UDCA reversed the increase in SFA content—especially C16:0 and C18:0 levels—induced by HFD and increased PUFAs in serum while decreasing MUFAs in liver and BAT.

### UDCA and Its Derivatives are Positively Correlated With PUFA and Inhibit TG Synthesis and FFA Synthesis and Uptake While Enhancing FFA Oxidation

**Figure 4:**
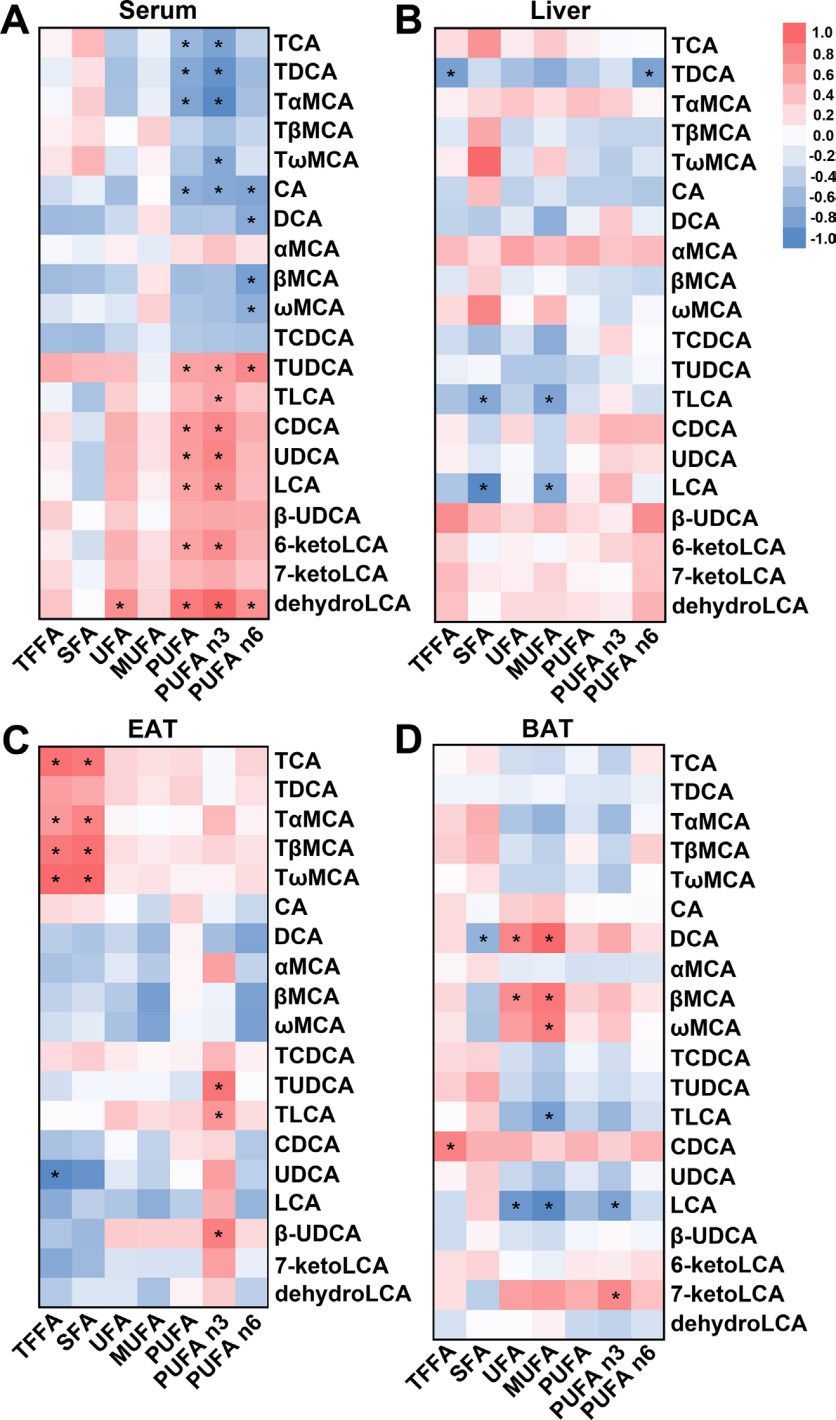
Correlation between BA and FFA species in different tissues. **(A**–**D)** Heat map based on correlation coefficients of BA and FFA in serum **(A)**, liver **(B)**, EAT **(C)**, and BAT **(D)**. *P < 0.05.

To clarify the relationship between BAs and FFAs, we evaluated the correlation between BAs that were markedly altered in the HFD and UDCA groups and FFA species. We found that the levels of UDCA and its derivatives including TUDCA, LCA, 6-ketoLCA, and dehydroLCA were positively correlated with serum PUFA content, and especially with n-3 PUFA concentration (**Figure 4A**). In contrast, TCA, taurodeoxycholic acid (TDCA), tauro-α-muricholic acid (TαMCA), and CA were negatively correlated with serum PUFA (**Figure 4A**). In the liver, TLCA and LCA levels were negatively correlated with SFA and MUFA contents (**Figure 4B**), which were decreased in the liver of the UDCA group (**Figure 3B**). Meanwhile, UDCA was negatively correlated with total FFA in EAT (**Figure 4C**). TCA and TMCAs contents—which were elevated by HFD (**Supplementary Figure 3C**)—were positively correlated with total FFA and SFA (**Figure 4C**). TUDCA, TLCA, and β-UDCA concentrations were positively correlated with that of n-3 PUFA (**Figure 4C**). TLCA and LCA were negatively correlated with MUFA (**Figure 4D**), which declined in BAT following UDCA administration (**Figure 3D**). Thus, UDCA increased PUFAs (especially n-3 PUFA), which could be due to the increased levels of UDCA and its derivatives, particularly TUDCA, TLCA, and LCA. On the other hand, the decreases in MUFAs in the liver and BAT in UDCA-treated mice could be related to the increases in LCA and TLCA.

**Figure 5:**
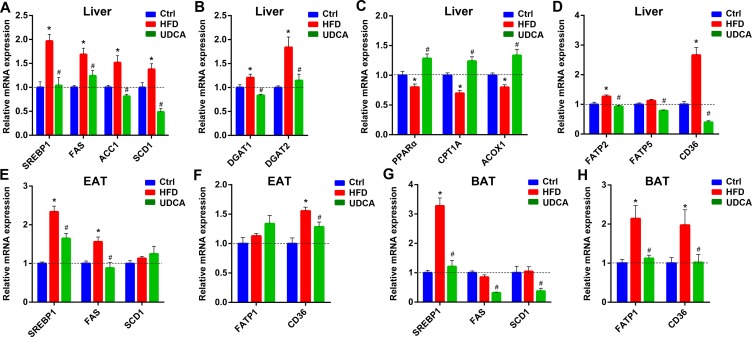
Expression of genes involved in FFA synthesis, uptake, and oxidation; and in TG synthesis in liver, EAT, and BAT after HFD and UDCA administration. **(A**–**D)** Relative mRNA expression of genes involved in FFA synthesis **(A)**, TG synthesis **(B)**, FFA oxidation **(C)**, and FFA transport **(D)** in liver. **(E**–**H)** Relative mRNA expression of genes involved in FFA synthesis in EAT **(E)** and BAT **(G)**, and involved in FFA transport in EAT **(F)** and BAT **(H)**. Ctrl, control group; HFD, 60% fat diet; UDCA, HFD with 0.5% UDCA. *P < 0.05 *vs*. control (ctrl) group; ^#^ *P* < 0.05 *vs*. HFD group.

To elucidate the mechanism by which UDCA alters FFA profiles *in vivo*, we examined the expression of genes involved in TG synthesis and FFA synthesis, oxidation, and uptake in liver, EAT, and BAT. Sterol regulatory element-binding protein 1c (SREBP1c)—a transcription factor that promotes the expression of lipogenic genes including acetyl coenzyme A (CoA) carboxylase 1 (ACC1), FA synthase (FAS), and stearoyl-CoA desaturase-1 (SCD1) (Horton et al., 2002)—was downregulated by UDCA treatment, with a corresponding decrease in *FAS*, *ACC1*, and *SCD1* mRNA levels (**Figure 5A**), indicating that *de novo* lipogenesis was inhibited. Diacylglycerol acyltransferase (DGAT) is the key enzyme promoting TG formation; we found here that UDCA suppressed *DGAT1* and *DGAT2* expressions (**Figure 5B**), which is consistent with the observed reduction in liver TG content (**Figure 1G**). The expression of factors involved in FA oxidation including peroxisome proliferator-activated receptor α (PPARα), carnitine palmitoyl transferase 1A (CPT1A), and acy-CoA oxidase-1 (ACOX1) was inhibited by consumption of HFD, but this was reversed by UDCA (**Figure 5C**). In addition, genes involved in FA uptake in liver including FA transport protein 2 (FATP2), FATP5, and cluster of differentiation 36 (CD36) were downregulated in the UDCA group, whereas FATP2 and CD36 were upregulated in the HFD group (**Figure 5D**). In EAT and BAT, factors associated with FA uptake and lipogenesis were also suppressed by UDCA administration to varying degrees (**Figures 5E–H**). These results demonstrate that UDCA alters the FFA profile by inhibiting lipogenesis, promoting FA oxidation, and reducing FA uptake in liver and adipose tissue. Additionally, UDCA repressed the expression of genes regulating TG synthesis, thereby decreasing TG deposition in liver.

## Discussion

In this study, we found that oral administration of UDCA reversed the metabolic dysfunction induced by consumption of HFD, as evidenced by the changes in BA and FFA profiles. The levels of UDCA and its derivatives were increased by UDCA administration. In particular, the level of TUDCA in the liver and adipose tissues was much higher than that of UDCA itself. Additionally, the level of TLCA—a downstream derivative of UDCA—was increased in these tissues. Meanwhile, UDCA treatment decreased the SFA content of most tissues while increasing that of PUFAs, especially n-3 PUFA. Moreover, the levels of UDCA and its derivatives were positively correlated with those of n-3 PUFA, TUDCA, and TLCA particularly. On the other hand, TLCA and LCA were negatively correlated with SFA in liver. Finally, we found that genes involved in *de novo* synthesis and uptake of FFA and TG formation were downregulated by UDCA administration, whereas genes related to FFA oxidation were upregulated. Thus, UDCA has the potential to improve the biochemical parameters associated with the development of obesity.

Studies have shown that UDCA has contradictory effects in metabolic diseases (Laurin et al., 1996; Lindor et al., 2004; Leuschner et al., 2010; Mueller et al., 2015). TUDCA, a UDCA derivative, was found to relieve endoplasmic reticulum stress and reduce leptin resistance (Ozcan et al., 2009) while the UDCA derivative GUDCA improved metabolic parameters as an antagonist of farnesoid X receptor (Sun et al., 2018). In this study, TUDCA level increased following UDCA treatment, suggesting that it is involved in the regulation of metabolism and alters FFA profile *in vivo*. UDCA was previously found to inhibit LCFA uptake by primary human hepatocytes in an FATP5-dependent manner (Nie et al., 2012), which is consistent with our finding that genes regulating FFA uptake in liver in UDCA-treated mice, as well as we found the similar result in EAT and BAT. In addition, we showed that TLCA was upregulated by UDCA administration, which was negatively correlated with SFA contents of liver. TLCA is an agonist of Takeda G protein-coupled receptor 5, a receptor that positively regulates energy metabolism (Chiang, 2013). The upregulation of genes involved in FA oxidation in the UDCA group may be related to the increase in TLCA. Meanwhile, the decrease in total liver FFA content could be attributed to the inhibition of FFA uptake and/or induction of FFA oxidation. Additional studies are needed to clarify the precise mechanism underlying the effects of UDCA on FA metabolism.

n-3 PUFA reduces inflammation and suppresses hepatic TG accumulation (Kim et al., 1999; Xu et al., 1999; Yahagi et al., 1999). In our study, we found that serum n-3 PUFA was increased following UDCA administration, which could contribute to the alleviation of metabolic dysfunction. In mice treated with UDCA, the FFA composition in BAT differed from that in other tissues, which was manifested as an increase in medium-chain FA proportion. Since FFA biosynthesis was inhibited in the UDCA group, this may have resulted from the decomposition of LCFA. We did not examine the expression of FA oxidation genes in BAT due to the limited amount of available BAT sample; however, the reason for the difference in BAT FFA composition warrants further investigation. Moreover, UDCA and TUDCA levels were higher in BAT than in EAT in the control group. This indicates that BA distribution varies across tissues, which could be associated with differences in organ function. This is the first study to report both BA and FFA profiles in EAT and BAT and the influence of UDCA on these profiles. An important next step will be to further clarify the function of UDCA and its derivatives and evaluate its safety and efficacy for the treatment of metabolic disease.

## Data Availability

The raw data supporting the conclusions of this manuscript will be made available by the authors, without undue reservation, to any qualified researcher.

## Ethics Statement

This study was carried out in accordance with the recommendations of the national legislation and local guidelines of the Laboratory Animals Center at Shanghai Jiao Tong University Affiliated Sixth Peoples Hospital, Shanghai, China. The protocol were reviewed and approved by the Institutional Animal Care and Use Committee at the Center for Laboratory Animals, Shanghai Jiao Tong University Affiliated Sixth Peoples Hospital, Shanghai, China.

## Author Contributions

YZ performed animal experiments, obtained BA and FFA profiles, analyzed the data, and drafted the manuscript. XZ, FH, and AZ participated in animal experiments. KG and QZ participated in measurement of BA and FFA levels. WJ was the project leader and designed the study.

## Funding

This work was supported by the National Key R&D Program of China (2017YFC0906800), National Natural Science Foundation of China (81772530 and 31500954), and International Science and Technology Cooperation Program of China (2014DFA31870).

## Conflict of Interest Statement

The authors declare that the research was conducted in the absence of any commercial or financial relationships that could be construed as a potential conflict of interest.

The handling editor declared a shared affiliation, though no other collaboration, with the authors at the time of review.

## Abbreviations

BA, Bile acid; FFA, free fatty acid; ITT, insulin tolerance test; OGTT, oral glucose tolerance test; TC, total cholesterol; TG, triglyceride; SREBP1c, sterol regulatory element-binding protein 1c; ACC-1, acetyl-coA carboxylase-1; FAS, fatty acid synthase; SCD-1, stearoyl-CoA desaturase-1; FATP1/2/5, fatty acid transport protein 1/2/5; PPARα, peroxisome proliferator-activated receptor alpha; CPT1A, carnitine palmitoyl transferase 1 A; ACOX-1, acyl-coenzyme A oxidase-1; DGAT1/2, diacylglycerol acyltransferase1/2; SFA, saturated fatty acid; UFA, unsaturated fatty acid; PUFA, polyunsaturated fatty acid; MUFA, monounsaturated fatty acid; CA, cholic acid; TCA, taurocholate acid; GCA, glycocholic acid; CDCA, chenodeoxycholic acid; TCDCA,taurochenodeoxycholic acid; GCDCA, glycochenodeoxycholic acid; α MCA, α-muricholic acid; Tα MCA, tauro-α-muricholic acid; β MCA, β-muricholic acid; Tβ MCA, tauro-β-muricholic acid; UDCA, ursodeoxycholic acid; TUDCA, tauroursodeoxycholic acid; GUDCA, glycoursodeoxycholic acid; LCA, lithocholic acid; TLCA, taurolithocholic acid; GLCA, glycolithocholic acid; DCA, deoxycholic acid; TDCA, taurodeoxycholate acid; GDCA, glycodeoxycholic acid; ωMCA, ω-muricholic acid; TωMCA, tauro ω-muricholic acid; dehydroLCA, dehydrocholic acid; isoLCA, isolithocholic acid; 6-ketoLCA, 6-ketolithocholic acid; 7-ketoLCA7-ketoLCA, 7-ketolithocholic acid; β-UDCA, 3β-Ursodeoxycholic Acid.
