# Supplementary material for: Ursodeoxycholic Acid Alters Bile Acid and Fatty Acid Profiles in a Mouse Model of Diet-Induced Obesity
Source: Front Pharmacol. 2019 Jul 25;10:842. doi: 10.3389/fphar.2019.00842 (PMC6669341; doi:10.3389/fphar.2019.00842)
Supplement: Supplementary file 1 [file DataSheet_1.pdf]

Supplementary materials

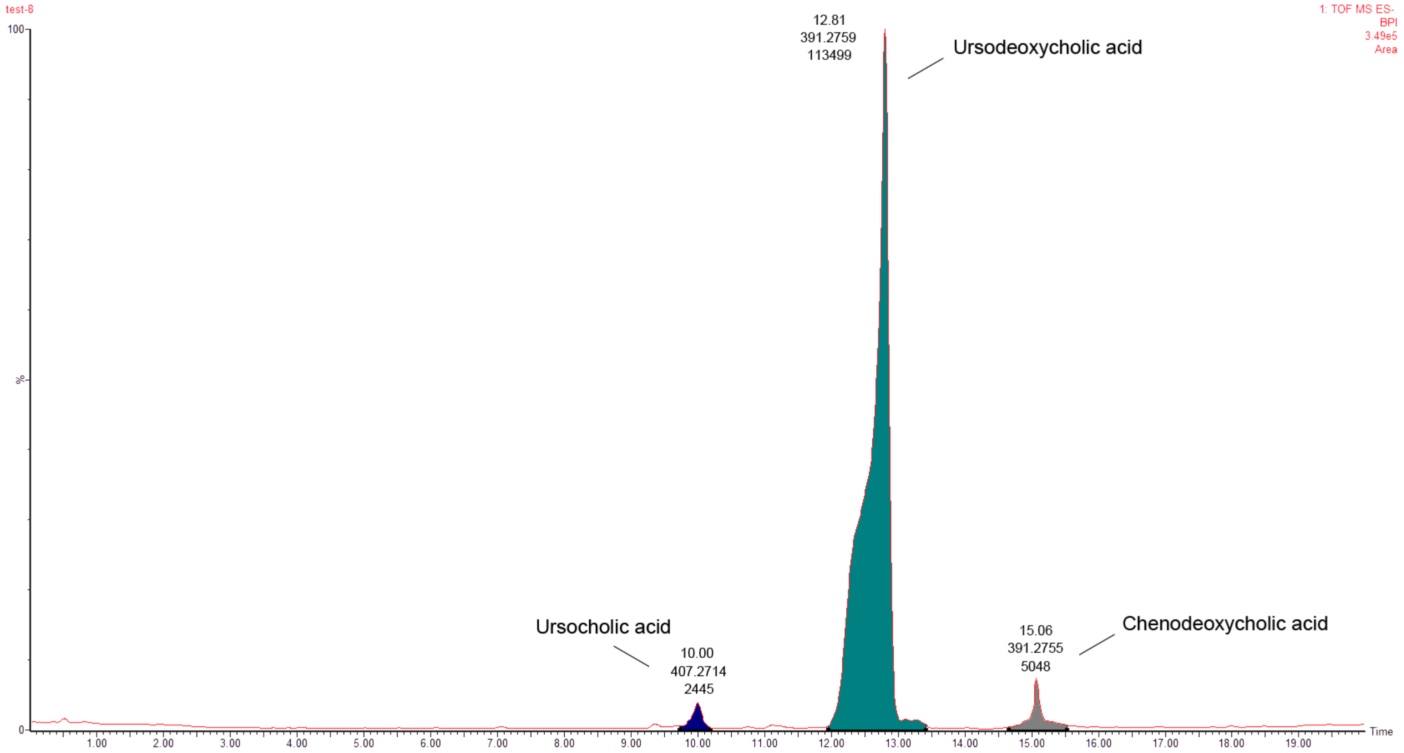

Supplementary Figure 1. Chromatograms of the dominating compounds in the drug UDCA that we used in the animal experiment.

The drug UDCA we used in the animal experiment was detected by UPLC-QTOF-MS, and we determined the purity of UDCA was about 94% according to the peak area, and two imputities were found, which were chenodeoxycholic acid (4%) and ursocholic acid (2%) respectively.

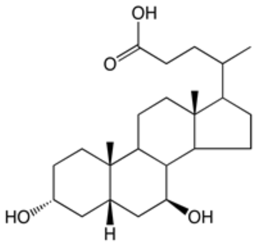

Supplementary Figure 2. The structure of UDCA.

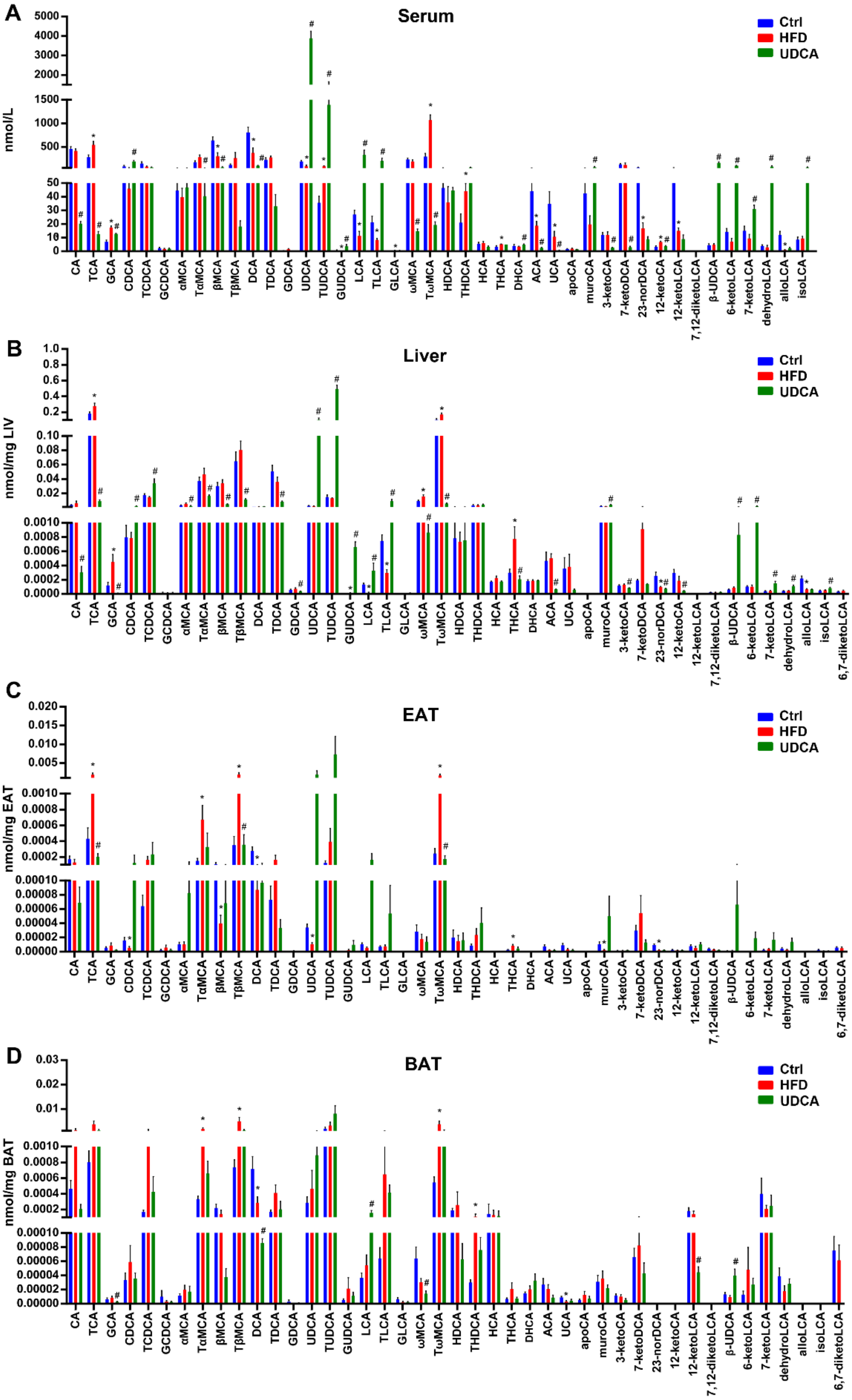

Supplementary Figure 3. BA concentrations in different tissues. (A–D) Concentration of individual BAs in serum (A), liver (B), EAT (C), and BAT (D). Ctrl, control group; HFD, 60% fat diet; UDCA, HFD with 0.5% UDCA. *P < 0.05 vs. control (ctrl) group; #P < 0.05 vs. HFD group.

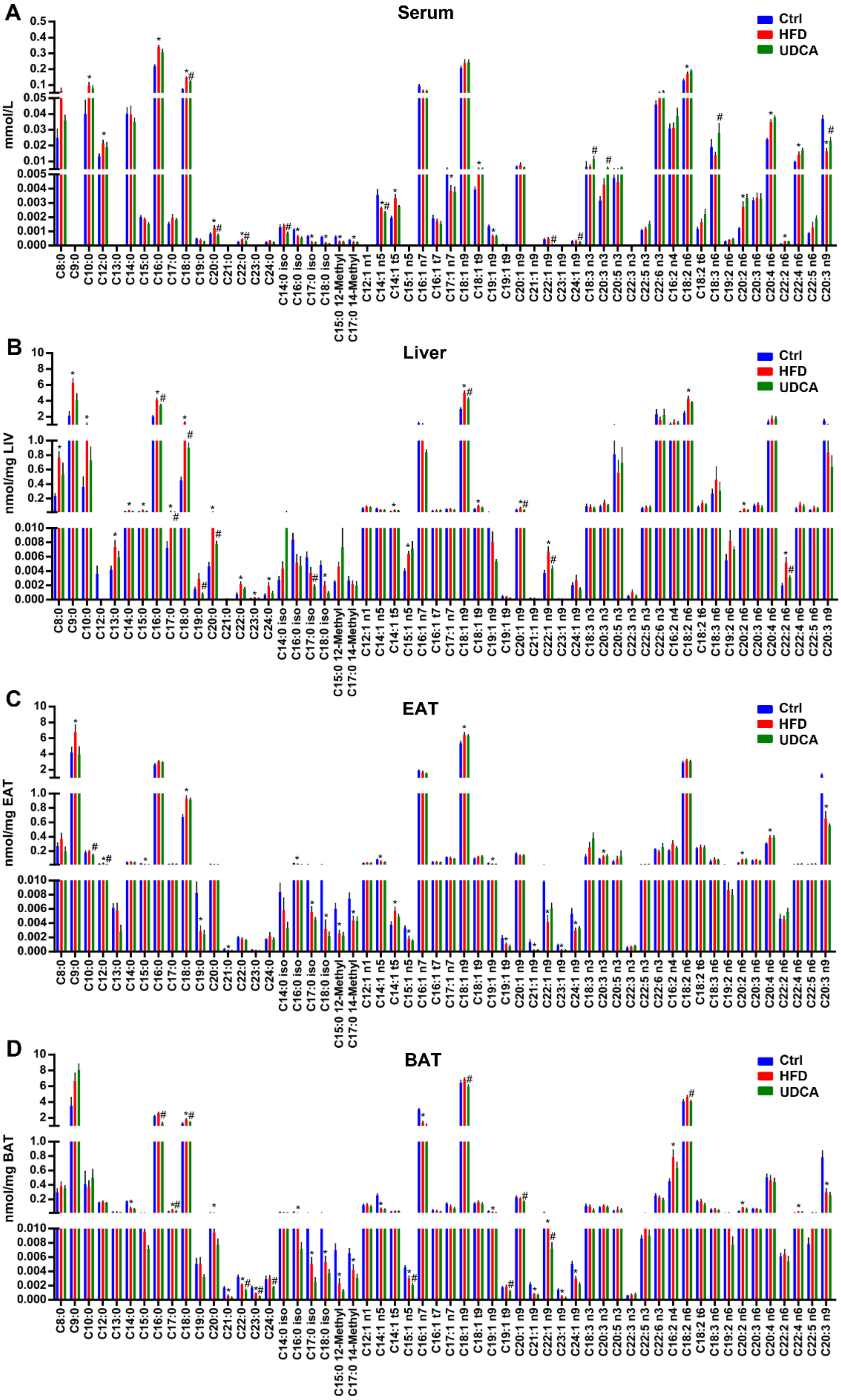

Supplementary Figure 4. Concentration of individual FFAs in different tissues. (A–D) Concentration of individual FFAs in serum (A), liver (B), EAT (C), and BAT (D). Ctrl, control group; HFD, 60% fat diet; UDCA, HFD with 0.5% UDCA. *P < 0.05 vs. control (ctrl) group; #P < 0.05 vs. HFD group.

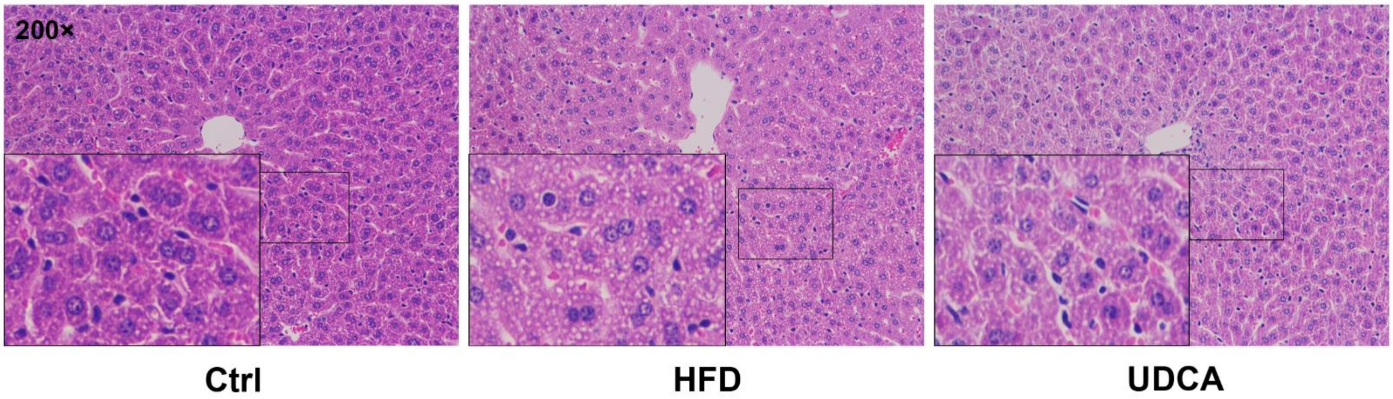

Supplementary Figure 5. HE staining of liver section of mice (200× magnification).

Supplementary Table 1. The gradient of mobile phase for bile acids detection

Gradient	%A	%B	Flow (mL/min)	Time (min)
1	80	20	0.45	0.00
2	80	20	0.45	2.00
3	75	25	0.45	3.00
4	75	25	0.45	6.00
5	65	35	0.45	8.00
6	65	35	0.45	11.50
7	1	99	0.45	18.00
8	1	99	0.45	19.00
9	80	20	0.45	19.10
10	80	20	0.45	20.00

Supplementary Table 2. The parameters of mass spectrometer for bile acids detection

Bile acids	Full name	Parent m/z	Daughter m/z
CA	cholic acid	407.35	407.35
TCA	taurocholate acid	514.35	79.93
GCA	glycocholic acid	464.35	73.93
CDCA	chenodeoxycholic acid	391.35	391.35
TCDCA	taurochenodeoxycholic acid	498.41	79.93
GCDCA	glycochenodeoxycholic acid	448.41	73.94
α-MCA	α-muricholic acid	407.35	407.35
Tα-MCA	tauro α-muricholic acid	514.35	79.93
β-MCA	β-muricholic acid	407.35	407.35
Tβ-MCA	tauro β-muricholic acid	514.35	79.93
UDCA	ursodeoxycholic acid	391.35	391.35
TUDCA	tauroursodeoxycholic acid	498.41	79.93
GUDCA	glycoursodeoxycholic acid	448.41	73.94
LCA	lithocholic acid	375.35	375.35
TLCA	taurolithocholic acid	482.41	79.93
GLCA	glycolithocholic acid	432.41	73.94
DCA	deoxycholic acid	391.35	391.35
TDCA	taurodeoxycholate acid	498.41	79.93
GDCA	glycodeoxycholic acid	448.41	73.94
ω-MCA	ω-muricholic acid	407.35	407.35

Tω-MCA	tauro ω-muricholic acid	514.35	79.93
HDCA	hyodeoxycholic acid	391.35	391.35
THDCA	taurohyodeoxycholic acid	498.41	79.93
GHDCA	glycohyodeoxycholic acid	448.41	73.94
HCA	hyocholic acid	407.35	407.35
THCA	taurohyocholic acid	514.35	79.93
GHCA	glycohyocholic acid	464.35	73.93
7-ketoDCA	7-ketodeoxycholic acid	405.35	405.35
ACA	allocholic acid	407.35	407.35
DHLCA	dehydrocholic acid	373.3	373.3
alloLCA	allolithocholic acid	375.35	375.35
isoLCA	isolithocholic acid	375.35	375.35
23-norDCA	23-nordeoxycholic acid	377.35	377.35
6-ketoLCA	6-ketolithocholic acid	389.35	389.35
7-ketoLCA	7-ketolithocholic acid	389.35	389.35
12-ketoLCA	12-ketolithocholic acid	389.35	389.35
apoCA	apocholic acid	389.35	389.35
muroCA	murocholic acid	391.35	391.35
β-UDCA	3β-ursodeoxycholic acid	391.35	391.35
6, 7-diketoLCA	6,7-diketolithocholic acid	403.35	403.35
3-ketoCA	3-dehydrocholic acid	405.35	405.35
7, 12-diketoLCA	7,12-diketolithocholic acid	403.35	403.35
12-ketoCA	12-ketocholic acid	405.35	405.35
UCA	ursocholic acid	407.35	407.35
β-CA	3β-cholic Acid	407.35	343.3
isoDCA	isodeoxycholic acid	391.35	391.35
DHCA	dehydrolithocholic acid	401.35	401.35
TDHCA	taurodehydrocholic acid	508.41	79.93
GDHCA	glycodehydrocholic acid	458.35	73.93
d4-CA		411.35	411.35
d4-LCA		379.41	379.41
d4-UDCA		395.41	395.41
d4-GCA		468.41	73.94
d4-GDCA		452.41	73.93
d4-GCDCA		452.41	73.93

Supplementary Table 3. The gradient of mobile phase for free fatty acids detection

Gradient	%A	%B	Flow (mL/min)	Time (min)
1	30	70	0.4	0
2	30	70	0.4	2.0
3	25	75	0.4	5.0
4	20	80	0.4	10.0
5	10	90	0.4	13.0
6	0.5	99.5	0.4	16.0
7	0.5	99.5	0.4	21.0
8	30	70	0.4	22.5
9	30	70	0.4	24.0
